# Chance Fluctuations in mRNA Output in Mammalian Cells

**DOI:** 10.1371/journal.pbio.0040319

**Published:** 2006-09-12

**Authors:** Françoise Chanut

In the drama of cell biology, both genetics and environment write the script, and chance throws in the twists of plot. In general, most cells live relatively predictable lives: divide, differentiate, and die. Yet chance leaves its imprint even in ordinary cells. For instance, bacterial or yeast cells in culture are known to produce widely different amounts of certain proteins, even when they are genetically identical. Scientists attribute such cell-to-cell variations to chance fluctuations in the cells’ ability to make these proteins. They also speculate that such fluctuations may benefit the cells in their struggle to adapt and survive.

But opinions vary as to which step in protein production is subject to random fluctuations. Proteins result from the translation of messenger RNAs (mRNAs), which come from the transcription of genes. Fluctuations in protein output could reflect a whimsy intrinsic to the expression of the coding gene, or random swings in global, or extrinsic, regulators of the gene’s expression. In yeast, extrinsic causes, such as variations in cellular volume, seem to predominate. But in a new study, Arjun Raj and his colleagues show that in mammalian cells, intrinsic causes, specifically the genes’ ability to transition randomly between active and inactive transcriptional states, can be crucial.

The researchers developed a sensitive technology to detect mRNAs in single cells and examined the output of a genetically engineered reporter gene they called M1, which they had introduced into Chinese hamster ovary (CHO) cells. After staining cultures of identical cells with a fluorescent probe specific to M1, they counted fluorescent dots corresponding to single M1 mRNA molecules in individual cells. At any given time, a few cells displayed a large, bright cluster of these spots, indicative of recently transcribed mRNAs still densely packed around the M1 gene. These cells also had the largest number of M1 mRNAs (over 150). In these cells, the M1 gene was therefore actively churning out mRNAs. But the majority of cells displayed fewer than 50 M1 mRNAs that were dispersed over their entire volume. In these cells, the M1 gene had become silent. These results showed that the M1 gene is expressed via infrequent bursts of transcriptional activity in the CHO cells.

If these bursts reflected the uneven distribution of transcription factors among CHO cells, then multiple reporter genes in a single cell should burst at the same time. The researchers generated cell lines that contained two versions of the reporter gene (M1 and M2) that were controlled by the same transcriptional activators but whose mRNAs could be distinguished with probes of different colors. In some lines, the two genes had landed in separate genomic locations. The researchers found that they burst independently of each other. They concluded that bursts are not induced by extrinsic factors such as M1- or M2-specific transcriptional activators. Rather, they reflect the intrinsically random ability of both the M1 and M2 genes to switch between an inactive and an active state.

**Figure:**
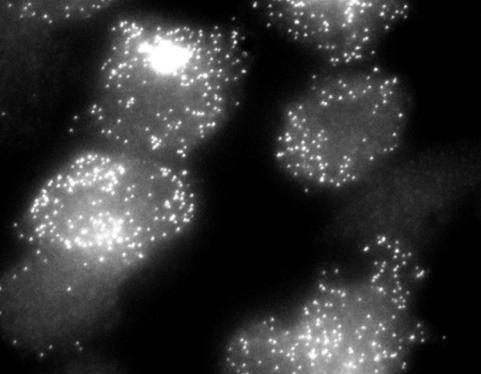
Individual reporter mRNA molecules are shown in a field of clonal CHO cells. Though genetically identical, the cells display extreme variations in their expression levels.

Nevertheless, when the two versions of the reporter gene landed next to each other, they did burst in synchrony, as if switching spread locally among contiguous genes. This observation suggests that switching follows the waves of condensation and decondensation that randomly breathe through the coils of genomic DNA (chromatin). Indeed, genes are mostly silent when packed into dense chromatin. But when they decondense, the transcription machinery can latch on to their DNA and begin making RNA. Raj and his colleagues propose that genes switch to an active state as a consequence of randomly initiated decondensation, and that transcription factors merely stabilize their active state. Consistent with this proposal, they find that reducing the availability of a transcriptional factor, or increasing M1’s affinity for this factor, does not significantly increase the frequency of bursts. Only the size of the bursts—that is, the amount of RNA produced in each burst—increases.

Random bursting is not restricted to artificial genes such as M1. The researchers document the same behavior in the gene encoding RNA polymerase, the lead actor of transcription. That cells would tolerate chance fluctuations in an mRNA so fundamental to their survival comes as a surprise. The researchers show that in the case of the M1 gene, protein stability buffers the consequences of erratic mRNA production. This is because stable proteins are only “topped up” by the occasional bursts of transcription, whereas unstable proteins follow the variations in mRNA levels more closely. This finding indicates that protein stability may be a critical factor in the cell’s ability to tolerate variations in transcription. Whether chance fluctuations in RNA production sometimes produce beneficial twists of fate for mammalian cells remains to be shown.

